# Hypochondriacal attitudes comprise heterogeneous non-illness-related cognitions

**DOI:** 10.1186/1471-244X-12-173

**Published:** 2012-10-17

**Authors:** Michael Schwenzer, Klaus Mathiak

**Affiliations:** 1 Department of Psychiatry, Psychotherapy and Psychosomatic Medicine, RWTH Aachen University, Pauwelsstr. 30, D-52074, Aachen, Germany; 2 JARA-Brain, Jülich Aachen Research Alliance, Translational Brain Medicine, Forschungszentrum Jülich GmbH: Section of Structural and Functional Organisation of the Brain (INM-1), Institute of Neuroscience and Medicine, Research Centre Jülich, Wilhelm-Johnen-Strasse, 52425, Jülich, Germany

**Keywords:** Hypochondriacal attitudes, Social fears, Self-esteem, Warm glow effect, Safety signals, Physician-patient relationship

## Abstract

**Background:**

Hypochondriacal attitudes were associated with cognitions not related to illness: Social fears, low self-esteem, and reduced warm glow effect, i.e. less positive appraisal of familiar stimuli. Only a single study had investigated the correlation of hypochondriacal attitudes with the warm glow effect so far and the present study aimed to corroborate this association. Particularly, the present investigation tested for the first time whether social fears, low self-esteem, and reduced warm glow effect represent distinct or related biases in hypochondriacal attitudes.

**Methods:**

Fifty-five volunteers filled in the Hypochondriacal Beliefs and Disease Phobia scales of the Illness Attitude Scales, two scales enquiring social fears of criticism and intimacy, and the Rosenberg Self-Esteem Scale. The interaction of valence and spontaneous familiarity ratings of Chinese characters indicated the warm glow effect.

**Results:**

A stepwise regression model revealed specific covariance of social fears and warm glow with hypochondriacal attitudes independent from the respective other variable. The correlation between low self-esteem and hypochondriacal attitudes missed significance.

**Conclusions:**

Hypochondriacal attitudes are embedded in a heterogeneous cluster of non-illness-related cognitions. Each social fears and a reduced cognitive capacity to associate two features – positive appraisal and familiarity - could diminish the susceptibility to safety signals such as medical reassurance. To compensate for reduced susceptibility to safety signals, multifocal treatment and repeated consultations appear advisable.

## Background

Hypochondriacal attitudes are a challenge for the health care system. Persons with hypochondriacal attitudes suffer from fears to be ill, thus their quality of life is clearly reduced [1,2]. Moreover, hypochondriacal attitudes accompany social conflicts with the medical staff and expensive re-examinations [3,4]. The cited findings included persons who were not diagnosed according ICD-10 [5] or DSM-IV [6]. The clinical manuals ICD-10 and DSM-IV define the most severe end of the spectrum [7] in terms of a cluster of symptoms persisting six month. However, a single hypochondriacal symptom could be sufficient to induce a clinically relevant disorder. For instance, disease phobia is associated with frequent attendance to primary care [8]. Thus, the general practitioner may want to start treating hypochondriacal attitudes even though not all criteria of ICD-10 or DSM-IV have been verified to prevent distress and aggravation [8,9]. To disentangle hypochondriasis on the subsyndromal level, the Diagnostic Criteria for Psychosomatic Research specified particular symptoms such as health anxiety or disease phobia [7]. As the following survey will show, there are reasons to consider further symptoms in hypochondriasis.

Knowledge about characteristic cognitions may help to identify hypochondriacal tendencies and to cut short the way to appropriate treatment [9]. Many studies on cognition in hypochondriasis and health anxiety were focused on illness-related biases, e.g. overrated dangerousness of bodily changes or sensations [10]. Non-illness-related cognitive biases, however, received poor attention although the ineffectiveness of medical reassurance as listed in clinical and subclinical classifications (criterion C in ICD-10 [5], criterion B in DSM-IV [6], the Hypochondriacal Beliefs scale of the Illness Attitude Scales [11,12]) suggests a social component. Mistrust in health professionals can neutralize the reassurance not to be ill [4,13]. Testing an interpersonal model of hypochondriasis, participants with hypochondriacal attitudes reported problems to deal with social relationships [13]. Hypochondriacal attitudes were associated with social fears of criticism and intimacy [14] which may contribute to the reduced trust in the safety signals from medical practitioners.

In studies on cognitive processes, a bias emerged towards both illness-related and social cues: Participants with hypochondriacal attitudes interpreted not only ambiguous sentences about health but also ambiguous sentences about social relationships more negative [15]. A negativistic bias independent of content suggested a deviant general cognitive process in hypochondriacal attitudes: Hypochondriacal attitudes were associated with less positive ratings of meaningless but familiar stimuli compared to unfamiliar stimuli [16]. The positive rating of familiar stimuli reflects the warm glow effect, i.e. mental association of familiarity with positive appraisal [17-19]. In another study, the response time in a Stroop task, i.e. to name the color of colored words as fast as possible, decelerated independent of the words’ meaning after experimentally induced illness concerns in participants who were preoccupied with illness [3]. A common reduction in cognitive processing may explain reduced warm glow and decelerated Stroop responses: In hypochondriacal attitudes the cognitive capacity to process two features - positivity and familiarity; words and colors - may be low. Conceivably, a limited cognitive capacity can explain under-weighing information about absent issues compared to the processing of reassuring facts in hypochondriacal attitudes as well [20]: To consider and dismiss information at the same time may complicate cognitive processing. Then again, a limited cognitive capacity may correlate with the neglect of safety signals and induce mistrust as a common theme: Mistrust in other people, in the own person and body’s functions, and in objects. Biases in several domains due to a single cognitive process such as reduced warm glow appears plausible because trans-categorical cognitive effects have been found elsewhere [21,22].

The association between a general cognitive bias and mistrust appears particularly evident in the negative evaluation of familiar stimuli in the reduced warm glow effect [16]. However, the correlation of reduced warm glow effect with hypochondriacal attitudes has been tested only once which is sparse evidence. Therefore, we consider its mere replication as an important contribution to hypochondriasis research. In addition, the present study tested for a potential correlation of reduced warm glow and social fears in hypochondriacal attitudes which to our knowledge had never been investigated before.

Hypochondriacal attitudes and social fears may be related to low self-esteem as well: Self-esteem may be affected by social fears [23] and low self-esteem was associated with hypochondriacal attitudes [24,25]. Hypochondriacal complaints may be aimed at distracting from personal failure [26] to set social fears and self-doubt at rest. However, a not so strong association between fear of criticism and low self-esteem [27] may challenge this model.

In summary, the review of literature suggested three potential predictors of hypochondriacal attitudes: Social fears, low self-esteem, and reduced warm glow effect. The present study was focused to continue the authors’ previous research on social fears and the warm glow effect [14,16]. Further potential variables such as obsession proneness [28] or magical thinking [29] in hypochondriasis would have been out of the scope of the present paper and should be considered in future research. So far, there is a complete lack of knowledge whether social fears and low self-esteem correlate with the reduced warm glow effect. Correlations between social fears, low self-esteem, and reduced warm glow effect either may provide an indication of a common bias or may help to disentangle hypochondriacal symptoms.

Measuring the associations of hypochondriacal attitudes with social fears, low self esteem, and reduced warm glow effect relied on established methods in the present study. The Hypochondriacal Beliefs and Disease Phobia scales of the Illness Attitude Scales assessed hypochondriacal attitudes [11,14,30]. Social fears were enquired with the same questionnaire as in [14]: the Unsicherheitsfragebogen [31]. Unlike [23] and [27] the present study applied the Rosenberg Self-Esteem Scale [32] because this questionnaire is well-known. For assessment of the warm glow effect, i.e. the interaction of appraisal and familiarity, the participants rated Chinese characters as in Zajonc’s study [33] about the mere exposure effect. However, the impression of familiarity was not induced by repeated exposure but was expected to emerge in some Chinese characters spontaneously during single presentation [16].

The present study presupposed that correlations of hypochondriacal attitudes with social fears, low self-esteem, and reduced warm glow effect could be replicated. The novel contributions were the analyses whether social fears, low self-esteem, and reduced warm glow effect predict hypochondriacal attitudes in equal measure and whether the predictors correlate with each other. The controversial hypotheses were tested that social fears, low self-esteem, and reduced warm glow effect constitute a common bias vs. they co-vary independently from each other with hypochondriacal attitudes.

## Methods

### Participants

Fifty-five volunteers (31 men and 24 women, ages 22 – 55 yrs., M = 31.9 ± 10.9 yrs.) from a sample of 106 subjects - students, employees, and visitors of the RWTH campus - were included. Inclusion criteria were no knowledge about Chinese characters and rating at least one Chinese character as familiar to assess the warm glow effect. In addition, the participants had to be free of disease according to a medical anamnesis to avoid a classification of physically-founded complaints as hypochondriacal. Ten volunteers afflicted with disease such as unclear gastro-intestinal complaints and cancer in remission, and 41 volunteers who rated no Chinese character as familiar were excluded from analysis. The sample size of 55 participants was appropriate to detect a large effect of the pivotal predictors of hypochondriacal attitudes in a multiple regression analysis with a power of about .8 [34]. No volunteer had participated in a similar study before; the participants differed from the sample of Schwenzer & Mathiak [16]. The ethical committee of the RWTH Aachen hospital approved the investigation. Participation was in accord to informed consent. However, the purpose of the Chinese test was not disclosed to get unbiased responses.

### Measures

Age, knowledge about Chinese characters, the medical anamnesis, and participation in previous studies were enquired in an interview. Familiarity ratings of Chinese characters (see the description below) were examined post-hoc.

Sum scores of two scales of the Illness Attitude Scales - Hypochondriacal Beliefs and Disease Phobia - indicated hypochondriacal attitudes [11,16,30]. Each scale presents three hypochondriacal attitudes, e.g. ‘Do you believe that you have a physical disease but the doctors have not diagnosed it correctly?’. The participants rated the occurrence of each attitude on a scale from ‘No’ (0) through ‘Most of the time’ (4). Sirri et al. [12] reported a good test-retest reliability between .4 and 1.0 and high correlations to other measures related to hypochondriasis (.5 < *r* < .8). According to Kellner [11], these two scales can identify clinical hypochondriasis.

Two scales of the Unsicherheitsfragebogen assessed social fears: Fear of Criticism and Fear of Intimacy [31]. Each scale consists of 15 statements to be rated from ‘Not at all’ (0) through ‘Totally correct’ (5). An example for an item on the Fear of Criticism scale: ‘It bothers me when others watch me working’. The Fear of Intimacy scale presented e.g. the item ‘It is always hard to start a conversation with a stranger’. The test-retest reliability of the scales is above .7; high scores in the Fear of Criticism and Fear of Intimacy scales correlate with social phobia [31].

The Rosenberg Self-Esteem Scale [32] assessed self-esteem by means of 10 items, e.g. ‘I feel that I have a number of good qualities’. No item regards social relationships with the exception of item four ‘I am able to do things as well as most other people’ which implies a comparison with other persons. Each item had to be rated from ‘Strongly agree’ through ‘Strongly disagree’. The answers were scored from 0 through 3 depending whether the item was a positive or negative statement about self-esteem. A low sum score indicates low self-esteem. The test-retest reliability was .85 and Rosenberg reported that low self-esteem was associated with psychosomatic symptoms, the expectation to be criticized by others, and low ability to criticize oneself [32].

For assessing a general cognitive bias, the participants rated 12 Chinese characters whose meaning they did not know. The number of Chinese characters was higher compared to a previous study [16] to increase the chance to find participants who rated at least on Chinese character as familiar for analyzing the warm glow effect. The Chinese characters were randomly chosen from an internet dictionary ( http://www.chinalink.de). The participants received a sheet with both a written instruction and the task on it. The instruction conveyed that Chinese characters based on pictograms and that the present test investigated people’s implicit understanding of pictorial meaning. For each character, the participants should guess whether the character described something good or something bad. The ratings were assessed using a semantic differential scale from good (0) through bad (6). The 12 Chinese characters were labelled from a through l. Below the Chinese characters their labels were written again. When a participant was under the impression that the character looks familiar to him, he marked the label assigned to the familiar character.

### Statistical analysis

The sum of scores from both hypochondriacal scales, the scores of each social and self-esteem scale, the mean of appraisal as well as the number of familiar Chinese characters, and the warm glow effect underwent analysis. In contrast to a previous study [14], we disregarded additional clinical cut-offs of the hypochondriacal attitudes and the social fears scales because few participants fulfilled these criteria and there was no comparable cut-off for the warm glow effect. Social anxiety can be regarded as trait which occurs in the non-clinical population and ranges on a continuum from mild discomfort to totally inhibiting anxiety [35,36]. Positive ratings and the number of familiar Chinese characters were analyzed separately because they may indicate a general negativistic bias or reduced familiarization. To simplify the presentation of the warm glow effect, a warm glow effect index was calculated: Mean ratings of familiar characters were subtracted from mean ratings of unfamiliar characters. A higher index score means a stronger warm glow effect.

To identify distinct associations between non-illness-related biases and hypochondriacal attitudes, a stepwise regression analysis was performed using the summed up Hypochondriacal Beliefs and Disease Phobia scores as dependent variable and fear of criticism, fear of intimacy, self-esteem, appraisal of Chinese characters, number of familiar Chinese characters, and the warm glow index as independent variables. The inclusion criterion was *p* < 0.050 two-tailed.

Means and standard deviations of each measure were differentiated for a low and high hypochondriacal group according to a median split of the summed up Hypochondriacal Beliefs and Disease Phobia scores to allow comparison with a previous study [16]. The same cut-off as in [16] (high hypochondriacal group > 1) and the Mann–Whitney-*U* test for analyzing group differences independent of data distribution were applied.

A further analysis investigated whether the non-illness-related cognitions which differed between the high and low hypochondriacal groups were associated with each other. The non-parametric Spearman’s coefficient that regarded non-Gaussian data distributions indicated correlations. In all analyses apart from the initial stepwise regression analysis, the significance level was *p* < .008 two-tailed according to the Bonferroni-correction of *p* < .050 for six variables that were potentially associated with hypochondriacal attitudes.

## Results

The stepwise regression model included fear of intimacy and reduced warm glow effect for the prediction of hypochondriacal attitudes; variance explained by both independent variables: *R*^*2*^ = 0.22, *F*(2,52) = 7.3, *p* = 0.002. Fear of intimacy: *B* = 0.08 ± 0.03, *β* = 0.30, *t* = 2.3, *p* = 0.023, *R*^*2*^ change = 0.146; warm glow effect: *B* = 0.44 ± 0.20, *β* = 0.28, *t* = 2.2, *p* = 0.031, *R*^*2*^ change = 0.074. Not to consider fear of intimacy produces a two-predictor model as well: *R*^*2*^ = 0.200, *F*(2,52) = 6.8, *p* = 0.002; warm glow effect: *B* = 0.52 ± 0.19, *β* = 0.33, *t* = 2.6, *p* = 0.009, *R*^*2*^ change = 0.137; fear of criticism: *B* = 0.06 ± 0.03, *β* = 0.26, *t* = 2.1, *p* = 0.036, *R*^*2*^ change = 0.071. In the first regression model, fear of criticisms did not explain further unique covariance with hypochondriacal attitudes because of its high correlation with fear of intimacy. Correspondingly, hypochondriacal attitudes correlated with fear of criticism (*ρ* = −0.36, *p* = 0.006), fear of intimacy (*ρ* = −0.39, *p* = 0.003), and reduced warm glow effect (*ρ* = −0.37, *p* = 0.005).

**Figure 1:**
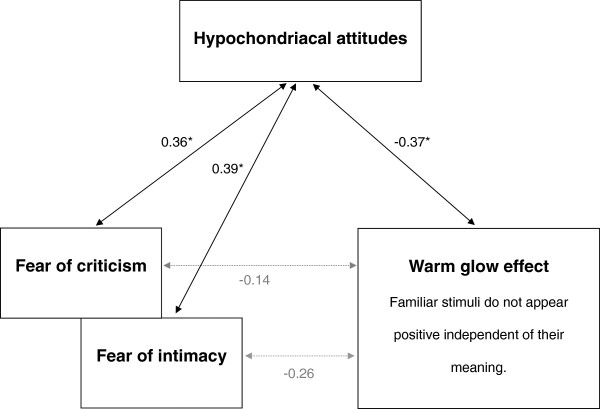
**Correlations between non-illness-related cognitions in hypochondriacal attitudes.** *p < 0.008 two-tailed.

Table 1 shows means and standard deviations of the low and the high hypochondriacal groups. Participants of the high hypochondriacal group reached higher scores in the social fears scales (each of both scales *Z* = 3.1, *p* = 0.001) and the warm glow effect was reduced in comparison to the low hypochondriacal group (*Z* = −3.1, *p* = 0.001). Ratings of familiar Chinese characters (mean appraisal in the high hypochondriacal group: *M* = 2.4 ± 1.2; low hypochondriacal group: *M* = 1.3 ± 1.2, *Z* = 2.9, *p* = 0.003) but not of unfamiliar Chinese characters (mean appraisal in the high hypochondriacal group: *M* = 2.8 ± 0.4; low hypochondriacal group: *M* = 2.9 ± 0.4, *Z* = 0.9, *p* = 0.333) were less positive in the high hypochondriacal group. Reduced warm glow did not correlate with fear of criticism (*ρ* = −0.14, *p* = 0.290) but showed a trend to be associated with fear of intimacy (*ρ* = −0.26, *p* = 0.035). Figure 1 illustrates that the correlation of social fears with the warm glow effect is lower than the correlations of each of them with hypochondriacal attitudes. Fear of criticism, fear of intimacy and low self-esteem (|*ρ*| > 0.50, *p* < 0.001) correlated with each other. Moreover, low self-esteem tended to correlate with reduced warm glow (*ρ* = −0.27, *p* = 0.040). Positive appraisal of Chinese characters and the number of familiar characters did not correlate with any other measure ( |*ρ*| < 0.16, *p* > 0.240).

**Table 1:** **Social fears, self-esteem, and general cognitive bias (*M*** **±** ***SD*) in a low and a high hypochondriacal group**

	**Low hypochondriacal**	**High hypochondriacal**
	*N* = 28	*N* = 27
**Social fears**		
Fear of criticism	28.1 ± 6.7	36.4 ± 10.0*
Fear of intimacy	27.1 ± 5.9	33.9 ± 7.8*
**Self-esteem**	34.0 ± 3.6	31.4 ± 3.5
**General cognitive bias**		
*-ratings of Chinese characters-*		
Positive appraisal	2.7 ± 0.4	2.7 ± 0.3
Number of familiar characters	1.8 ± 0.9	1.6 ± 0.7
Warm glow effect	1.6 ± 1.1	0.3 ± 1.4*

Note: *p = 0.001 two-tailed.

## Discussion

The study confirmed the correlation between hypochondriacal attitudes and reduced warm glow effect [16] a second time and thus this finding appears reliable. Moreover, social fears and reduced warm glow effect were associated independently from each other with hypochondriacal attitudes. Thus, social fears and reduced warm glow effect constitute two distinct symptoms. Though the cross-sectional design cannot demonstrate causal directions, some models appear more likely than others due to the present data. Conceivably, concerns to be ill induce fears of social failure and take away cognitive capacities such that hypochondriacal persons cannot focus on two additional features such as positivity and familiarity of objects. From this a vicious cycle may start: Since the patient is preoccupied by illness concerns he can hardly process safety signals such as medical reassurance ↔ since he hardly is aware of safety signals the illness concerns persist. This model assumes that it is more likely that one cause (illness concern) induces two independent consequences (social fears and reduced cognitive capacity) than vice versa. Experiences with illness during childhood could plausibly explain illness convictions [37].

The low correlation between social fears and reduced warm glow effect does not fit in a general bias in terms of mistrust or impaired familiarization. A self-serving function of hypochondriacal complaints [26] does not appear plausible as well. The low correlation of self-esteem with hypochondriacal attitudes is compatible with moderate statistical significance in much larger samples [24,25] meaning secondary relevance. The combination of high correlations with social fears and low correlation with self-esteem indicates that persons with hypochondriacal attitudes attribute conflicts rather to an external cause than to their self-concept [4]. This is in line with another finding that persons with hypochondriacal attitudes attend to social relationships [38]. However, preoccupation with the body and the reduced warm glow effect indicate that external orientation is not limited to social relationships in hypochondriacal attitudes.

The lack of negative appraisal of unfamiliar Chinese characters supports neither an amplifying bias [39], i.e. persons with hypochondriacal attitudes exaggerate the interpretation of sensations including basic sensory perceptions, nor a general pessimistic view. The reduced warm glow effect in meaningless Chinese characters may rather be related to decelerated naming the colors of colored words independent of the words’ meaning suggesting reduced cognitive processing [3,40]. The potential causes of familiarity can be neglected in the present study because the frequency of familiarity per se did not interact with hypochondriacal attitudes.

The findings may have clinical implications. Social fears and a reduced warm glow effect may predict a difficult physician-patient relationship and a higher probability of unnecessary medical examinations. It is purposeful to specify relevant biases in a non-clinical sample to optimize the assessment of the rare patients according to ICD-10 or DSM-IV. The present data suggest that hypochondriasis is under-determined without inclusion of non-illness-related criteria. Particularly as the evidence of superior and stable success due to illness-focused treatment was questionable [41]. It is invalid to consider a social approach ineffective [42] when therapy studies skipped the assessment of social issues [42-44]. On the other hand, restricted susceptibility to safety signals due to social fears and reduced cognitive capacity suggest the importance of repeated consultations in the clinical management of patients with hypochondriasis. This gives an empirical foundation for de Zwaan and Müller’s recommendation [45] to increase the frequency of consultations in hypochondriacal disorders. A higher number of consultations increase the probability to recognize safety signals. Besides, de Zwaan and Müller [45] suggest a time and not a symptom contingent schedule to avoid reinforcement of complaints. This conclusion agrees with Kellner’s work on explanatory therapy for hypochondriacal fears and beliefs, which was based on adequate medical reassurance, information, and explanation after an accurate medical evaluation [46]. A disorder of general cognitive processing - the reduced warm glow effect - independent of social fears suggests that hypochondriasis could be disentangled from anxiety disorders and its treatment may rely on an additional mechanism compared to the therapy of fears. The present findings encourage investigating social fears and reduced cognitive capacity in a future study including patients with hypochondriasis diagnosed according ICD-10.

## Conclusions

Hypochondriacal attitudes are embedded in a heterogeneous cluster of non-illness-related cognitions: Social fears and reduced warm glow effect, i.e. less positive appraisal of familiar stimuli. Each social fears and a reduced cognitive capacity to associate two features – positive appraisal and familiarity - could diminish the susceptibility to safety signals such as medical reassurance in hypochondriacal attitudes. Thus, multifocal treatment and repeated consultations appear advisable.

## Competing interests

The authors declare that they have no competing interests.

## Authors’ contributions

MS conceptualized the study, analyzed the data, and drafted the manuscript. KM made substantial contributions to the statistical analysis, interpretation of data, and revising the manuscript. Both authors read and approved the final manuscript.

## Pre-publication history

The pre-publication history for this paper can be accessed here:

http://www.biomedcentral.com/1471-244X/12/173/prepub
